# Photoelectrochemical Enzyme Biosensor for Malate Using Quantum Dots on Indium Tin Oxide/Plastics as a Sensing Surface

**DOI:** 10.3390/bios14010011

**Published:** 2023-12-24

**Authors:** Tereza Hlaváčová, Petr Skládal

**Affiliations:** Department of Biochemistry, Faculty of Science, Masaryk University, Kamenice 5, CZ-62500 Brno, Czech Republic; tereza.hlavacova@recetox.muni.cz

**Keywords:** malate dehydrogenase, flow-through biosensor, electrochemical sensor, wine, fruit juice

## Abstract

A photoelectrochemical biosensor for malate was developed using an indium tin oxide (ITO) layer deposited on a poly(ethylene terephthalate) plastic sheet as a transparent electrode material for the immobilization of malate dehydrogenase together with CdTe quantum dots. Different approaches were compared for the construction of the bioactive layer; the highest response was achieved by depositing malate dehydrogenase together with CdTe nanoparticles and covering it with a Nafion/water (1:1) mixture. The amperometric signal of this biosensor was recorded during irradiation with a near-UV LED in the flow-through mode. The limit of detection was 0.28 mmol/L, which is adequate for analyzing malic acid levels in drinks such as white wines and fruit juices. The results confirm that the cheap ITO layer deposited on the plastic sheet after cutting into rectangular electrodes allows for the economic production of photoelectrochemical (bio)sensors. The combination of NAD^+^-dependent malate dehydrogenase with quantum dots was also compatible with such an ITO surface.

## 1. Introduction

Since Becquerel’s discovery of the photoelectric effect in 1839, photoelectrochemistry (PEC) has been constantly developing. Nowadays, it is a multidisciplinary area involving optics, electrochemistry, surface science, and solid-state physics [1,2]. During the PEC process, the energy transfer from light to suitable acceptors within the electrochemical interface occurs after photons are absorbed by the photoactive material—a semiconductor. Electron–hole pairs are formed, causing an oxidation–reduction reaction and thus electron exchange with molecules leading to the change in the photocurrent [2,3]. Two possible mechanisms for the generation of a photocurrent exist. The first one is the presence of a reducing agent; in this case, the excited states are reduced to the ground state, and the PEC reaction can be triggered again. The second type is the presence of donor and acceptor molecules, wherein the transfer of electrons occurs between excited and quenching molecules; the resulting state of the molecule—reduced or oxidized—allows it to further exchange electrons with the electrode surface and generate a photocurrent, and the photoactive material returns to the ground state [3].

A PEC cell contains a working electrode—a semiconductor immobilized on the surface of the transparent conductive oxide—and an auxiliary electrode. The working electrode is connected to the auxiliary electrode via an electrolyte that cannot interfere with the analyte. In typical PEC biosensors, PEC active material and a biological recognition element are required; a tris(bipyridyl) ruthenium complex and CdS quantum dots (QD) frequently serve as photoactive components [4,5,6].

PEC concepts are applied for immunoassays and DNA analyses and especially in combination with enzymes. In PEC enzymatic biosensing, the enzyme itself or the components participating in enzyme conversion have to communicate with the PEC active species for the effective transfer of electrons [4]. In principle, the change in electric signal is measured during the enzymatic reaction with the analyte. Typically, the change in current corresponds to the concentration of the analyte. Enzyme-based PEC analysis can be divided into three groups based on the mechanism of the electron transfer between the enzyme and the electrode. In the first group, techniques mainly involving the use of oxidoreductases and the consumption of an electroactive co-substrate or the formation of an electroactive co-product are followed. For example, the redox-active cofactor pair NAD^+^/NADH is necessary for more than 300 enzymatic reactions [6,7,8]. The second group relies on the presence of a mediator—a small redox-active molecule with the ability to transfer electrons between the active site of the enzyme and the electrode. Mediators are in most cases organometallic molecules such as ferrocenes, ruthenium or osmium complexes, and quinones. The last PEC enzymatic bioanalysis group is based on direct electron transfer between enzymes (for example, those with prosthetic groups such as hems and cytochromes) and an electrode. Furthermore, combination with nanomaterials has been found to be beneficial for direct electron transfer [6].

For working electrodes in PEC systems, transparent conductive oxides (TCO) are often chosen as semiconductive materials with high optical transparency and high electrical conductivity [1]. The first TCO thin film was prepared in 1907 by oxidizing a film of cadmium metal; CdO is not used today due to its toxicity. In 1937, the first tin oxide thin film was prepared, and, in 1954, a conductive thin film of indium oxide was developed. Both tin and indium oxide films are made via the post-oxidation of thin metal films. The most notable TCO in past years is tin-doped indium oxide (ITO) [9,10]. ITO is a solid solution of In_2_O_3_ and SnO_2_, typically in a ratio of 9:1.17. There are several techniques for the production of thin films on substrates such as glass or PET [5]. ITO films are fabricated using electron beam evaporation, ion-assisted plasma evaporation, direct current/radio frequency magnetron sputtering, thermal evaporation, pulsed laser deposition, chemical vapor deposition, or chemical solution deposition [11]. Low-sheet-resistance thin ITO films can be prepared via vacuum-based vapor deposition, but the production costs are higher [12]. The solution-based techniques, namely, inkjet, spray, and dip coating, are simple and quite cheap; however, to reduce sheet resistance, several depositions are required [13]. Overall, using ITO films as electrode materials can be cheaper than using gold or platinum electrodes [14].

ITO is a semiconductive material with a band gap between 3.5 and 4.3 eV and low electrical resistivity. It has unique properties, such as transparency to visible and near-infrared light, a wide electrochemical working window, good electrical conductivity, good substrate adhesion, low capacitive current (typically), and reasonable chemical inertness [9,15]. Therefore, ITO is widely used for technological and scientific applications such as liquid crystal and flat panel displays, heat-reflecting mirrors, electroluminescent devices [15], transparent electrodes used in photovoltaic instruments [16], absorbents for gas sensors [17], and coatings for electrodes for the fabrication of biosensors [12,15].

The surfaces of ITO electrodes are commonly modified using nanoparticles (NPs) for improving properties such as electric conductivity and sensitivity; the use of Au NPs [18,19] and carbon nanotubes [13] has been reported. Quantum dots are fluorescent colloidal semiconductor nanocrystals widely used due to their special photophysical properties such as size-controlled luminescence, high fluorescence quantum yield, and excellent stability against photobleaching since their discovery in 1980s [20,21]. The color of the luminescence can be controlled by the duration of the growth of QDs and the resulting size and quantum confinement effect. When the size of a particle is too small to be comparable to the wavelength of the electron, quantum confinement is observed, and this effect depends on the material and its band gap energy. With a decreasing size of the nanocrystal, the energy of the band gap increases. To enhance photoluminescence, a core quantum dot (QD) can be coated with a layer of a higher band gap energy semiconductor to form a core–shell structures [22,23]. This shell protects the core from photobleaching and oxidation. Upon illumination of the QDs, electron–hole pairs are generated, and recombination processes in solution result in light emission. The immobilization of the QDs onto electrode surface results in a longer lifetime of the electron–hole pair. This allows for electron transfer to the electrode resulting in an increase in the PEC current [21,24]. Functional groups can be used to modify the surface of the QDs, increasing their hydrophilicity and making them better suited for labeling of biomolecules [25] and for enzyme-linked PEC bioanalysis [26,27].

Malate dehydrogenase (MDH) is an oxidoreductase that is found in various cell compartments and catalyzes the reversible oxidation of malate to oxaloacetate using the NAD^+^/NADH cofactors. There are several isoenzymes that can be distinguished based on their specificity towards either NAD^+^ or NADP^+^. In eukaryotic cells, two main isoenzymes can be found: one in mitochondria as part of the citric acid cycle and the other in the cytosol participating in the malate-aspartate shuttle [28]. MDHs are typically found as dimers or tetramers consisting of identical subunits with weights ranging from 30 to 35 kDa. In solution, the MDHs are stable as homodimers. Each subunit contains two functionally distinct domains: the NAD^+^ binding site located on the amino-terminal half of the molecule and the substrate binding site located on the carboxy-terminal half. The active site is located in the cleft between these domains [29]. Kinetic studies have shown that NAD^+^ binds first and it is followed by malic acid. After the formation of the ternary complex enzyme–coenzyme–substrate, the protein undergoes a conformation change and the external loop closes over the active site to protect the substrate and important residues from the surrounding solution [30]. Both oxalacetate and reduced cofactor are eventually released.

Recently, a review [31] discussed the combination of various dehydrogenases, including MDH, with different types of nanomaterials, including QDs. In this study, we combined CdTe core-type QDs with MDH from the thermophilic bacterium *Thermus flavus* to photoelectrochemically determine malic acid in drinks by detecting NADH formed during the enzymatic reaction.

## 2. Materials and Methods

### 2.1. Materials and Chemicals

NaH_2_PO_4_∙2H_2_O, NaHCO_3_, NaOH, NaCl, acetic acid, MDH (*Thermus flavus)* and gelatin were purchased form Penta (Prague, Czech Republic). Glutaraldehyde (GA), bovine serum albumin (BSA), chitosan, QDs CdTe core type, malic acid, Nafion and polyethyleneimine (PEI) were purchased from Sigma-Aldrich. The ITO-PET (poly(ethylene terephthalate) bulk sheets (1 × 1 ft size, 930 cm^2^) of resistances 60, 100 and 250 Ω/sq were also purchased from Sigma-Aldrich. H_2_O_2_ was obtained from P-Lab (Prague, Czech Republic). K_3_[Fe(CN)_6_] was purchased from Lachema (Brno, Czech Republic). Water used in experiments was purified using the Milli-Q system. Phosphate-buffered saline (PBS) (50 mmol/L NaH_2_PO_4_, 150 mmol/L NaCl, pH 7.0) was prepared in the laboratory.

### 2.2. Instrumentation

Amperometry and cyclic voltammetry were performed by EmStat (Palmsens, Houten, The Netherlands) either in stirred beaker or in a flow-through cell of own construction. Magnetic stirrer (IKA, Staufen, Germany) was used for amperometric measurements in first part of this work. pH of solutions was determined by a pH meter (WTW inolab, Burladingen, Germany). Peristaltic pump Minipuls MP3 (Gilson, Villiers-le-Bel, France) with silicone tubes also from Gilson was used for amperometric measurements with the enzyme. Photoelectrochemical measurements were realized with different types of LEDs (green 525 nm, blue 470 nm, UV 395–400 nm and white) operated under stabilized working current. The working current for each LED was kept within the lower half of the maximum allowed operating current. The individual LED currents were adjusted using the Fiber Optic Spectrometer 2048XL (Avantes, Apeldoorn, The Netherlands) to achieve equal photon counts. The electrochemical current response signal was then measured at 500, 700 and 1200 mV applied to the ITO electrode, in plain PBS and with NADH.

### 2.3. ITO-PET Based Electrodes

ITO-PET was cut to strips with size 0.5 × 3 cm and the strips were partially masked with an adhesive tape to define the size of the active measuring surface, which was equal to 0.25 cm^2^ during modifications and measurements. The pseudo reference electrode used was Ag/AgCl and a Pt sheet served as counter electrode. For the initial electrochemical characterization, we used either 1 mmol/L H_2_O_2_ or 1 mmol/L K_3_[Fe(CN)_6_] solutions in PBS for cyclic voltammetry (CV) within a potential range of −1 V (start) to 1 V at a scan rate of 100 mV/s. We also tested ITO-PET amperometrically at −0.6 V in a flow-through cell sealed with rubber o-rings. K_3_[Fe(CN)_6_] solutions (1, 3, and 5 mmol/L) were tested at a potential of −0.6 V, and the flow rate was 1, 3, and 5 mL/min for the injected solutions for 0.5 and 1 min periods. Similarly, H_2_O_2_ solutions (1, 3, and 5 mmol/L) were tested at −0.7 V. Additionally, NADH was measured at 0.3, 0.5, 0.7, 0.8, and 1.2 V using concentrations of 0.1 and 1 mmol/L.

The QDs CdTe@ITO-PET electrodes were obtained by modifying the ITO-PET strips. The chitosan solution used was 200 µL 0.5% in 1% acetic acid and the QDs suspension of 100 µL had a concentration of 5 mg/mL. Two immobilization approaches were tested for the QDs: (1) Deposition of QDs onto ITO-PET followed by overlapping the surface with a solution of chitosan in acetic acid after drying. (2) Preparation of a mixture of QDs and chitosan (1:1) and application onto the ITO-PET surface. The resulting layers were gently rinsed with water and stored in a dry state after overnight drying.

### 2.4. Immobilization of MDH and QDs on ITO-PET

To prepare the immobilization mixtures, stock solutions of MDH (10 mg/mL in PBS), BSA (50 mg/mL in PBS), GA (3% in water), QDs (5 mg/mL), gelatin (18% in PBS) and PEI (18% in water) were used. The strips with the deposited mixtures were refrigerated overnight. The following day, the strips were gently rinsed with water, allowed to dry, and stored in the refrigerator until use.

- **Crosslinking with GA**. A 40 µL mixture was prepared by combining 10 µL of MDH, 10 µL of BSA, 20 µL of PBS and 3 µL of GA. Next, 10 µL of the mixture was immediately applied on the QDs@ITO-PET.
- **Gelatin** solution was warmed in a water bath, and an immobilization mixture was prepared by combining 40 µL of MDH, 10 µL of BSA, 20 µL of PBS, 20 µL of gelatin and 30 µL of QDs. This mixture was quickly deposited on the ITO-PET.
- **Gelatin/GA** immobilization process was the same as the previous one. The immobilization mixture was composed of 40 µL of MDH, 10 µL of BSA, 20 µL of PBS, 20 µL of gelatin, 30 µL of QDs and 5 µL of GA.
- **Polyethylene imine**. The immobilization mixture consisted of 20 µL of PEI, 40 µL of MDH, 10 µL of BSA, 20 µL of PBS and 30 µL of QDs. 10 µL of the mixture was applied onto the ITO-PET strip and allowed to dry.
- **PEI/GA** was nearly the same as above, with the addition of 2.5 µL of GA (in this case, 1% solution was used).
- **Nafion 1**. This mixture contained 5 µL of MDH, 1.25 µL of BSA, 2.5 µL of Nafion, 4.5 µL of QDs and 10 µL of PBS. 10 µL of the mixture was applied onto the surface of the ITO-PET.
- The other three variants **Nafion 2, 3 and 4** followed the same first step of immobilizing a mixture with the enzyme. The mixture used in these variants contained 20 µL of MDH, 5 µL of BSA, 13.5 µL of QDs and 10 µL of PBS. This mixture was also applied to the surface of ITO-PET and allowed to dry. The subsequent step involved coating the enzyme layer with 5 µL of Nafion at the initial concentration and diluting it with water in either a 1:1 or 1:2 ratio before allowing it to dry.
- **PEI/GA/Nafion**. The immobilization mixture consisted of 10 µL of MDH, 2.5 µL of BSA, 5 µL of PBS, 5 µL of PEI, 7.5 µL of QDs, and 1.25 µL of GA (1%). The mixture was applied to the ITO-PET strip and allowed to dry. Afterwards, 5 µL of Nafion diluted with water (1:1) was used to overlay the enzyme mixture.

### 2.5. Measurement Procedure with PEC Biosensors 

**Figure 1:**
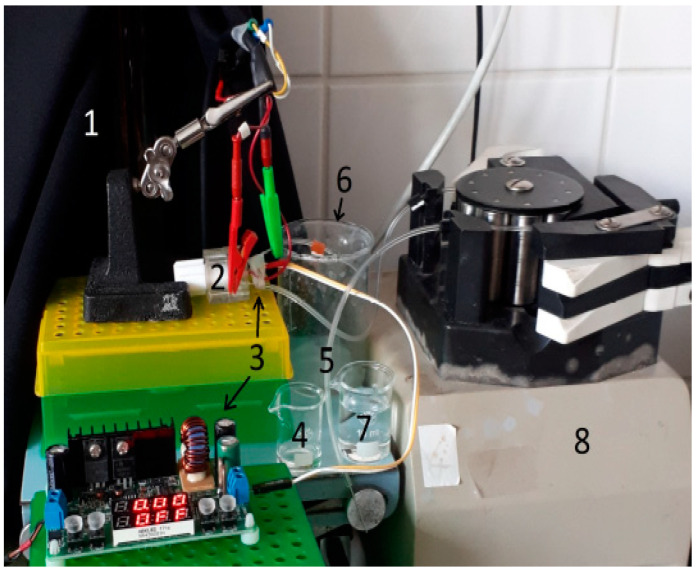
The experimental setup. 1—Black opaque cloth for covering the system, 2—flow-through cell with a ITO-PET sensor connected to a detector, 3—UV LED and its control system, 4—beaker with a substrate, 5—dosing silicone tube, 6—waste beaker, 7—beaker with PBS, and 8—peristaltic pump.

Strips with immobilized MDH were fixed into the flow-through cell and the whole system was sealed, the illumination LED was placed close to the surface of the biosensing layer. To eliminate interference from daylight, the measuring system was covered with a black opaque cloth. Figure 1 shows the setup. 

The peristaltic pump was set to a flow rate of 3 mL/min. The running buffer consisted of PBS containing 1 mmol/L NAD^+^, and pH was adjusted to 6.5. The samples of drinks with pulp were filtered to remove the coarse particles before analysis. The malate standards and analyzed samples were prepared using the same running buffer, with a constant final concentration of 1 mmol/L NAD^+^. The illumination periods consisted of 5 seconds in the buffer, followed by a dark period, and then another 5 seconds in the presence of either standard or sample zone.

## 3. Results and Discussion

**Figure 2:**
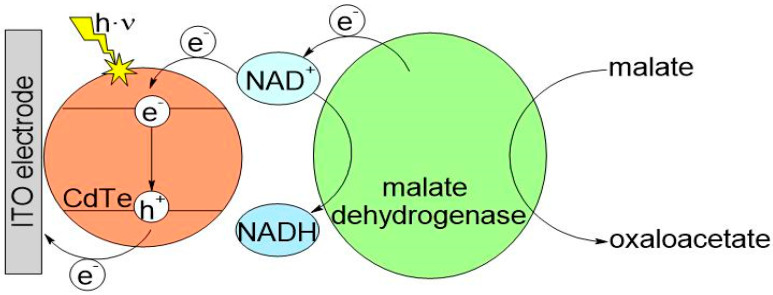
Electron transfer scheme in the enzyme-based photoelectrochemical biosensor.

The PEC biosensor concept is presented in Figure 2. To ensure efficient communication between the enzyme active site and the semiconducting QDs via the reduced cofactor NADH resulting from the enzymatic reaction, co-immobilization of CdTe QDs and malate dehydrogenase needs to be optimized.

### 3.1. Testing Properties of ITO-PET

In order to construct a photoelectrochemical enzyme-based biosensor, we utilized the ITO-PET material. The surface of the original ITO sensor was characterized using atomic force microscopy (Dimension FastScan Bio, Bruker, Santa Barbara, USA, dry state imaging), which revealed a surface roughness of approximately 30 nm. Following the deposition of rather thick enzyme/quantum dots mixed biolayers, the roughness increased significantly up to 210 nm. Electrochemical impedance spectroscopy characterization was performed. The impedance of bare ITO electrodes in the presence of 5 mM ferricyanide resulted in a charge transfer resistance *R*_CT_ = 35 ± 7 kΩ, different immobilization procedures increased this value by up to 5-fold.

Subsequently, the material was tested to determine its suitability as a working electrode. The bulk ITO-PET sheet obtained was cut into small pieces to serve as sensor strips. Cyclic voltammetry and amperometry were utilized to test the performance of ITO-PET with model redox-active substances including K_3_[Fe(CN)_6_], H_2_O_2_, and NADH, which is a product from the MDH reaction. The concentrations used were 1 mmol/L. The amperometric measurements of these substances yielded satisfactory results. The ITO-PET electrode provided lower current densities compared to common metal-based electrodes of similar area. However, it was found to be excellent for the electroconversion of ferricyanide providing 22 µA, and hydrogen peroxide, which peaked at 820 nA. Subsequently, the focus shifted to the electrochemical oxidation of NADH at the ITO-PET electrode. The results from these measurements were significantly lower—below 10 nA compared to either ferricyanide or hydrogen peroxide. Therefore, the ITO-PET alone is not a suitable alternative for the simplest direct amperometric detection of NADH. As a result, an alternative photoelectrochemical approach was adopted, which uses QDs on the electrode surface, to solve the problem (see Figure 2).

### 3.2. Testing the QD@ITO-PET Photoelectrochemical Properties

We attempted two different ways of immobilizing the QDs onto the ITO-PET surface. The first method involved applying a QDs suspension and overlaying it with 0.5% chitosan. The second method involved immobilizing of a mixture of QDs with chitosan, but no signal was detected upon illumination. Therefore, we proceeded with using the QDs@ITO-PET strips coated with chitosan.

**Figure 3:**
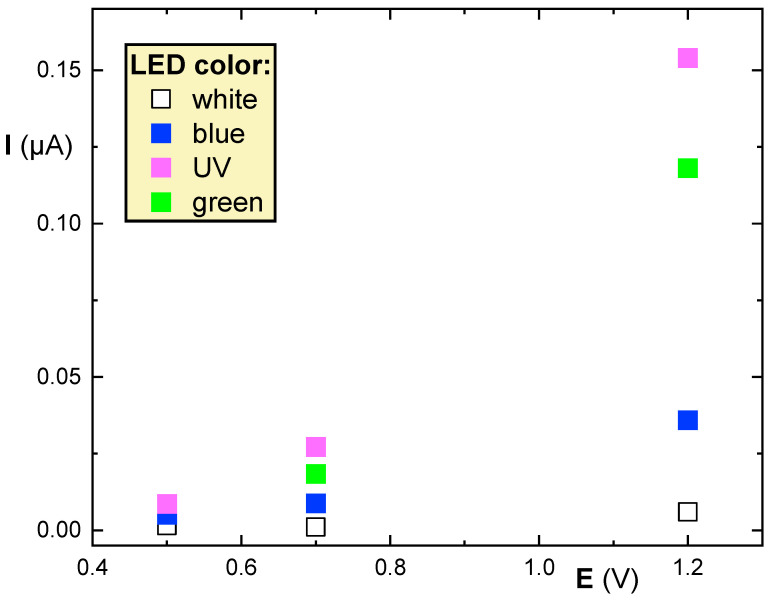
Comparison of signal of 0.2 mmol/L NADH upon illumination with different LEDs.

To illuminate the quantum dots, it was necessary to find a suitable light source. Amperometric measurements were carried out in solution of PBS and NADH with a concentration of 0.2 mmol/L. To prevent interference from daylight, the system was covered with black cloth. Four different LEDs were used to illuminate the QDs, and the photocurrent values were measured at 0.5, 0.7, and 1.2 V (see Figure 3).

Based on the responses presented in the reference, it is clear that the (near) UV LED was the most effective source for exciting electrons in QDs, and was therefore used in subsequent experiments. Reasonable responses were also obtained with green and blue LEDs, while the currents measured with the white LED were significantly smaller. It should be noted that the type 250 Ω/sq ITO-PET sheet was used consistently as it provided the best results. Responses from the 100 and 60 Ω/sq options were 3-fold lower and close to negligible, respectively.

### 3.3. Immobilization of MDH

The careful design of the enzyme biolayers should combine both experimental optimization with various modeling approaches to fine tune performance [32]. In this study, several types of malate biosensors were constructed using different procedures for immobilizing MDH. The microbial MHD enzyme isolated from *Thermus flavus* was used. All performance comparison measurements were carried out under the same conditions in a flow-through cell. NADH molecules were produced during the reaction of malate as a substrate with the enzyme. A signal was generated in the presence of 1 mmol/L NAD^+^ cosubstrate in the PBS solution.

**Figure 4:**
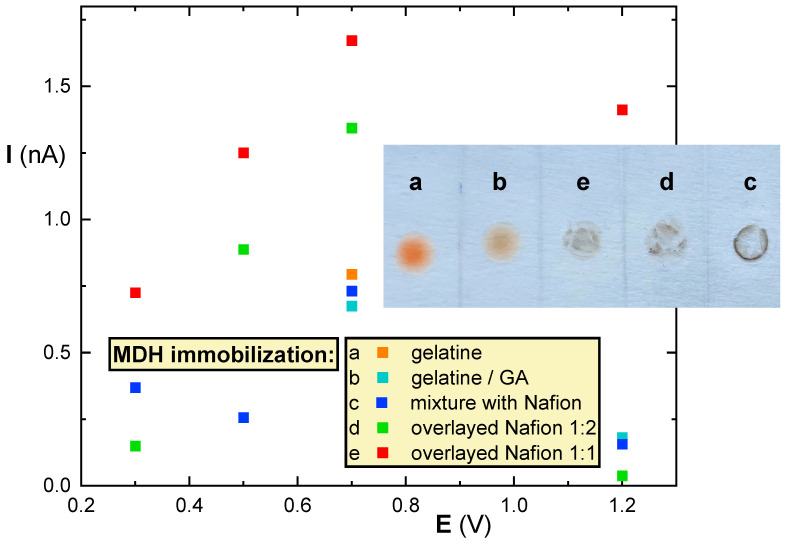
Comparison of the signals from different immobilizations (the labels a to e and the point colors are provided inside the figure) of MDH in the presence of 1 mmol/L malic acid. The inset image provides photos of the individual biosensing layers in dry states after completed immobilization procedures.

Several variations in suitable immobilization of MHD were tested. The first immobilization approach used 3% GA as a homobifunctional agent to connect the enzyme molecules through amino groups and capture QDs in the resulting reticulum. The immobilization of MDH in gelatin was tested with and without the use of a 3% GA. The immobilizations showed lower signals of NADH than in case of using the GA alone. As an alternative to gelatin, PEI was also tested, with one mixture containing GA and another without. The presence of PEI in the mixture without GA caused a significant color change in the QDs from orange to grey. This resulted in no NADH response, which was further complicated by the poor adhesion of the mixture to the ITO-PEI surface. However, the addition of GA did not improve the performance. The use of 3% GA was unsuitable for immobilization due to clotting, and therefore only 1% GA was used. However, a similar issue arose as in the previous approach. Initially, the quantum dots changed color, and the adhesion to the ITO-PET was poor and unstable. Subsequently, alternative immobilizations of MDH with Nafion were attempted and successfully evaluated (see Figure 4).

The mixture of the enzyme with Nafion and QDs produced a signal, but unfortunately, the signal peaks were too low to distinguish from background noise. The other variants, including the mixture of the enzyme with QDs overlayed with the stock Nafion solution, Nafion diluted with water in ratios of 1:1 and 1:2, gave signals correlating with the concentration of malate. Figure 4 provides images of the enzyme layers deposited on ITO-PET strips.

As shown in Figure 4, the highest signal was obtained when the mixture of MDH overlayed with Nafion diluted with water in a ratio of 1:1 ratio was immobilized. This was used for further work. The photographs of the immobilized biolayers indicate a nicely homogeneous deposition based on gelatin (a,b), the Nafion-based layers (c,d,e) resulted with higher surface inhomogenities. However, the measured response was preferred as a parameter for optimization. 

**Figure 5:**
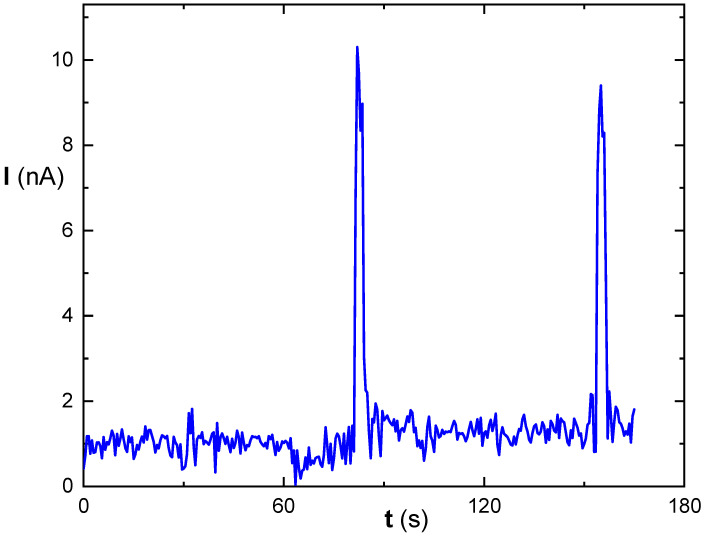
The example of measurement of signal in the presence of 0.1 mmol/L malic acid at 0.3 V using the biosensor with immobilized MDH mixture overlayed with Nafion diluted with water in a ratio of 1:1. Two subsequent peaks of current (=response) correspond to 5 s illumination periods (LED ON), and the background corresponds to the dark current (LED OFF).

Figure 5 shows the amperometric signal trace over time obtained from measuring using the immobilized mixture of MDH overlayed with Nafion diluted 1:1 with water. The relative low working potential was selected to minimize potential interferences, particularly from the planned measurements of real wine and fruit juice samples.

The pH optimum of free MDH from the organism *Thermus flavus* is 10.7 at a temperature of 30 °C and 9.5 at 60 °C. However, the immobilization procedure and the subsequent steps involved in the response may affect this optimum. Therefore, it was necessary to determine optimal pH for the overall response. Measurements were conducted at a laboratory temperature of 22 ± 2 °C.

**Figure 6:**
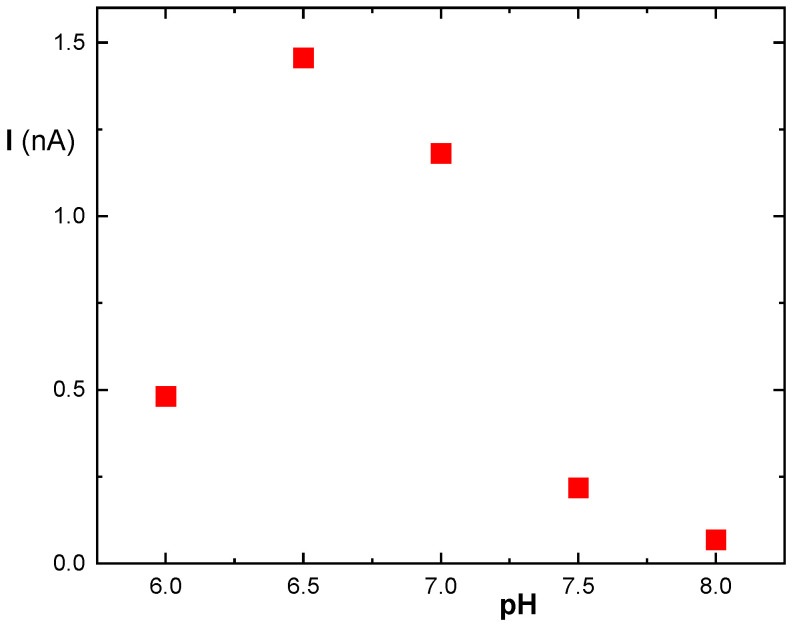
The dependence (red squares) of the MDH biosensor on pH. Malic acid with a concentration of 0.1 mmol/L, potential was set to 0.3 V.

**Figure 7:**
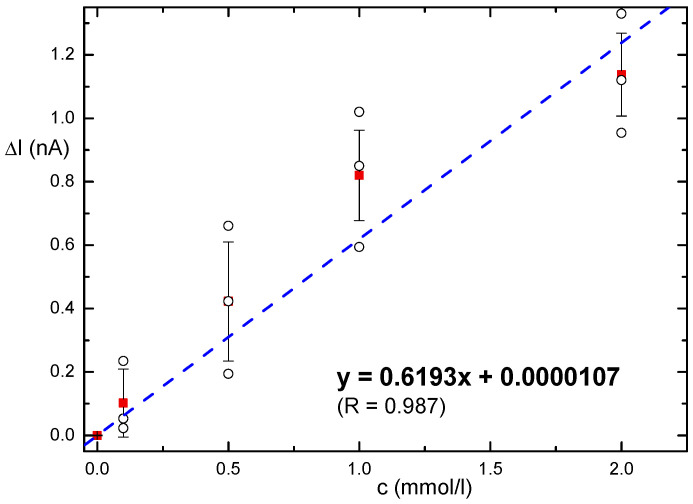
Calibration of the MDH biosensor using malic acid standards, plot of peak current minus the background dark current. For each average point (red filled rectangles), the error bar represents the standard deviation (n = 3, data from three individual biosensors were used, black empty circles).

Figure 6 shows a shift in the pH optimum towards the lower pH. The best signal was observed at pH 6.5, which was used to measure the concentration of malic acid in the samples. A calibration curve was constructed (see Figure 7). The concentration range up to 2 mmol/L malic acid was approximated with a straight line, the linear regression parameters are presented in the figure.

The limit of detection (LOD) of 0.28 mmol/L was estimated using the 3SD/slope of calibration approach based on the fluctuations of responses for the lowest tested concentrations from several biosensors (SD, standard deviation). Only one voltametric biosensor for malate based on immobilization of MDH using ITO was found. It is based on an octadecylamine-based Langmuir-Blodget film deposited on a classic ITO electrode [33]. This biosensor reported an LOD of 0.66 mmol/L. Table 1 summarizes the other purely electrochemical approaches. A direct comparison of the published approaches is challenging due to the significant influence of the chosen electrode material, immobilization method, and transduction procedure on performance. Additionally, a detailed review provided also information of several malate-specific enzymes suitable for biosensor construction [34]. Our photoelectrochemical approach provides a feasible alternative to amperometric sensors due to the cost-effective electrode material. The working range is appropriate for analyzing juice drinks and wines.

### 3.4. Determination of Malic Acid Concentration in Drinks

The performance of the biosensor in real samples was evaluated by determining the concentration of malic acid in drinks. To decrease the effect of interferences, simplify filtration of dense juices before filtration, and adjust pH, all samples were diluted 10 fold with the working buffer. Filtration was necessary to remove the pulp particles present in juices, but not required for wine samples. Five different fruit drinks and two white wines were tested using apple concentrate or apple puree. Each sample was measured five times using a new sensor. The biosensors were stored in a dry, dark refrigerator and provided 90% and 65% of the initial response after 1 and 3 months of storage, respectively.

Malic acid concentrations were determined and are presented in Table 2 after recalculation for dilution. Among the fruit drinks tested, Aquila Fruttimo had the lowest malic acid concentration. The label on the bottle indicates that it is a mixture of apple juice and water, and it is the only sample that indicated the addition of malic acid as an acidity regulator. On the other hand, Tesco 100% apple juice, which is supposed to be a native juice prepared directly from fresh apples, had the highest concentration of malic acid. In terms of wine samples, Irsai Oliver, made from green grapes, had a higher concentration of malic acid compared to Zweigelt Claret, a white wine made from red grapes, which had a substantially lower content. The sample with the highest error in determining malic acid concentration was Relax juice 100% apple, which had the highest relative standard deviation. In contrast, the sample with the lowest RSD was Tesco 100% apple, which was directly pressed. 

Three samples were selected to evaluate the recovery of spiking with added malic acid levels: Tesco 100% apple—directly pressed, Irsai Oliver, and Relax Exotica Mango. The samples were diluted 10 fold, and 0.5, and 1 mmol/L malic acid concentrations were added, mixed, filtered, and measured using the biosensors.

The measurement of malic acid concentration in spiked samples resulted in the best recovery values for the Tesco 100% apple sample (refer to Table 3). Acceptable recoveries were also determined for the chosen wine sample, Irsai Oliver. In this study, we analyzed the malic acid content in commercial wine samples. However, it is possible to foresee other applications in the field of wine production and monitoring of malolactic fermentation [39].

The Relax Exotica mango juice yielded significantly higher results than expected. The sample was initially dense and difficult to filter, which may have affected the biosensing layer due to the presence of small particles passing through the filtration adapter. Centrifugation could be considered as an alternative to filtration, but this would require laboratory analysis.

## 4. Conclusions

In this paper, we presented an economic approach to producing transparent electrodes. We found that commercially available large ITO/PET sheets can be cut to approximately 600 rectangular electrodes, resulting in a negligible price per sensor. The electrochemical and transparent properties of this material make it suitable for constructing photoelectrochemical biosensors for malic acid. Additionally, the low cost of the material allows for disposable use, which is important for analyzing complex biological samples. The use of plastics in the ITO layer support restricts the modification procedures. It is important to avoid harsh conditions such as the presence of organic solvents and extreme pH values, as they can cause a degradation of the sensing properties. In this case, the enzyme presence required mild deposition procedures based on buffers. The ITO strip electrode used was effective in the assays of malic acid in fruit juices, which often contain fruit pulp. The LOD can be expected to improve with the implementation of an automated procedure for biosensor preparation.

Additionally, the use of transparent electrochemical materials such as ITO/PET will enable more widespread application of combined electrochemical and optical measurements, which are useful for various research directions.

## Figures and Tables

**Table 1:** Comparison of L-malate electrochemical biosensor based on immobilized malate dehydrogenases.

Construction	Method	LOD/Upper Limit (mmol/L)	Ref.
glass electrode	pH sensor	0.75/30	[35]
gC, SWCNT, MDH in Nafion	amperometry	0.033/1.3	[36]
Au, MDH and DP in polypyrrole	amperometry	0.000063/0.001	[37]
Au, MWCNT, MDH and DP	amperometry	0.0016/0.61	[38]
ITO, Langmuir-Blodget film	voltammetry	3/50	[33]
ITO, MDH and QDs in Nafion layer	photoelectrochemical	0.28/2	this work

Abbreviations: DP diaphorase; gC glassy carbon; MWCNT multiwalled carbon nanotubes; SWCNT single-walled carbon nanotubes.

**Table 2:** Measurement of malic acid contents in real samples; for each sample, five measurements were used to obtain the average content and its standard deviation.

Samples	Malate (mmol/L)	SD (mmol/L)
**Fruit juices**		
Aquila Fruttimo apple	7.2	2.6
Relax juice 100% apple	14.2	8.5
Relax Exotica mango	10.5	4.2
Tesco 100% apple—directly pressed	24.1	3.4
Tesco 100% apple—purefruit	22.6	8.7
**Wines**		
Irsai Oliver	14.0	3.7
Zweigelt Claret	9.3	2.5

**Table 3:** Recovery of the malic acid concentrations—0.5 and 1.0 mmol/L—used for spiking of the selected fruit juices and wine sample. The 10-fold diluted samples were used in all cases.

Sample/Spiked Level (mmol/L)	Malate ± SD (mmol/L)	Recovery (%)
Tesco 100% apple—directly pressed/0	2.41 ± 0.34	-
Tesco 100% apple—directly pressed/0.5	2.95 ± 0.21	108
Tesco 100% apple—directly pressed/1.0	3.50 ± 0.29	109
Relax Exotica mango/0	1.05 ± 0.42	-
Relax Exotica mango/0.5	2.05 ± 0.29	200
Relax Exotica mango/1.0	3.26 ± 0.97	226
Irsai oliver/0	1.40 ± 0.37	-
Irsai oliver/0.5	2.06 ± 0.38	132
Irsai oliver/1.0	2.59 ± 0.34	119

## Data Availability

The data presented in this study are available on request from the corresponding author. The data are not publicly available due to further planned original processing procedures.
